# Experience of implementing and delivering group consultations in UK general practice: a qualitative study

**DOI:** 10.3399/BJGP.2020.0856

**Published:** 2021-03-09

**Authors:** Laura Swaithes, Zoe Paskins, Helen Duffy, Nicola Evans, Christian Mallen, Krysia Dziedzic, Andrew Finney

**Affiliations:** Impact Accelerator Unit, School of Medicine, Keele University, Keele.; Impact Accelerator Unit, School of Medicine, Keele University, Keele; NIHR clinician scientist, Haywood Academic Rheumatology Centre, Midlands Partnership Foundation Trust, Staffordshire.; Impact Accelerator Unit, School of Medicine, Keele University, Keele.; Impact Accelerator Unit, School of Medicine, Keele University, Keele.; NIHR research professor in general practice and head of the School of Medicine, Keele University, Keele.; National Institute for Health Research (NIHR) senior investigator, professor of musculoskeletal therapies, and director of the Impact Accelerator Unit, School of Medicine, Keele University, Keele; visiting professor, Faculty of Health and Applied Sciences, University of the West of England, Bristol; honorary implementation consultant, Midlands Partnership Foundation Trust; adviser to the National Institute for Health and Care Excellence Fellows and Scholars Programme.; Impact Accelerator Unit, School of Medicine, Keele University, Keele; senior lecturer of nursing, Clinical Education Centre, University Hospitals of North Midlands NHS Trust, Royal Stoke University Hospital, Stoke-on-Trent.

**Keywords:** barriers, general practice, primary health care, semi-structured interviews, shared medical appointments, implementation

## Abstract

**Background:**

Group consultations are a relatively new concept in UK primary care and are a suggested solution to current workload pressures in general practice. Little is known about the experience of implementing and delivering this approach from staff and organisational perspectives.

**Aim:**

To explore the experience of implementing and delivering group consultations in general practice.

**Design and setting:**

Qualitative telephone interview study.

**Method:**

Topic guides explored the perspectives and experiences of general practice staff on the implementation and delivery of group consultations. Data analysis adopted principles of the Framework Method underpinned by Normalisation Process Theory.

**Results:**

Interviews were conducted with 8 GPs, 8 practice nurses, 1 nurse associate, 1 practice pharmacist, 1 deputy practice manager, and 1 healthcare assistant. Four themes were identified: sense making of group consultations; the work associated with initiating group consultations; the experiences of operationalising group consultations; and sustaining change. Group consultations made sense to participants as a mechanism to reduce burden on primary care, enhance multidisciplinary working, and provide patient-centred care. Implementation required strong leadership from a ‘champion’, and a facilitator had a pivotal role in operationalising the approach. The associated workload was often underestimated. Barriers to embedding change included achieving whole practice buy-in, competing practice priorities, and system-level flexibility.

**Conclusion:**

General practice clinicians enjoyed group consultations, yet significant work is required to initiate and sustain the approach. An implementation plan considering leadership, roles and responsibilities, and wider organisational support is required at the outset. Further research or evaluation is needed to measure process outcomes.

## INTRODUCTION

UK general practice has been described as ‘at breaking point’ with concerns regarding current demand, efficiency of services, a recruitment and retention crisis, and staff ‘burn-out’.^1^^,^^2^ Innovative delivery models utilising a range of professionals and broader team working are suggested ways to address these pressures.^3^^,^^4^ Group consultations are one of the ‘Ten High Impact Actions’ for general practice to release capacity and reduce workload.^5^

Group consultations (or shared medical appointments) offer an alternative to one-toone consultations for long-term conditions (LTCs), providing clinical management, patient education, and peer support.^6^^,^^7^ Groups can be delivered face-to-face or online with up to 15 patients with the same condition.^8^ Consultations are co-delivered with at least one clinician (for example, GP, practice nurse [PN], or pharmacist) alongside a facilitator, lasting approximately 60–90 minutes.^9^ Delivery and format may vary but, in general, the facilitator is responsible for group preparation including a ‘results board’ of patient biometrics for discussion. The facilitator manages the group and supports patients to generate questions for the clinician, who joins the group to address questions collectively and provide personalised one-to-one discussion.

Evidence from systematic reviews and meta-analyses suggests that group consultations are effective in improving clinical outcomes, such as haemoglobin A1C (HbA1c) and blood pressure in diabetes, and are associated with positive patient experience measures, although questions remain about the impact on other clinical outcomes and the feasibility and cost-effectiveness of the approach.^10^^,^^11^ Although few studies have cited barriers to implementation,^12^ a lack of confidence and skills in facilitation, along with an ingrained, established model of consultation, are suggested reasons for hesitancy regarding the approach.^9^^,^^13^^,^^14^ However, a paucity of evidence exploring implementation in UK general practice exists.^6^

Future-proofing the primary care workforce requires service delivery models not only to be responsive to patient needs but also to be adaptable and acceptable to those who are delivering them. In the UK, group consultations are an emerging approach, with little known about the extent to which they are practised and the experiences of general practice services adopting them.^5^^,^^9^ To understand how to optimise implementation, it is therefore necessary to engage with stakeholders to identify and address potential implementation challenges from a staff perspective.^10^ The aim of this study was to explore the experience of implementing and delivering group consultations in primary care in the context of the NHS.

**Table:** How this fits in

Group consultations are a relatively new approach in UK general practice, with much of the literature examining the impact on clinical outcomes. This study explores the experiences of general practice staff implementing and delivering group consultations. Recommendations for clinicians focus on the need for an implementation strategy that considers local contextual circumstances, and, local and national evaluation data measuring process outcomes, to support the set-up and sustainability of the approach.

## METHOD

Telephone semi-structured interviews were used to understand the perspectives and experiences of primary care staff involved in the implementation and delivery of group consultations. This study was reported using the consolidated criteria for reporting qualitative research checklist.^15^

A multidisciplinary stakeholder advisory group, conducted with general practice staff, primary care academics, and public contributors, sought opinions of group consultations and informed topic guide development specific to each professional discipline. These were modified iteratively during the interviews, informed by field notes and author discussion, to test ongoing interpretations and examine anomalous responses (see Supplementary Appendix S1). Individuals (including GPs, pharmacists, PNs, healthcare assistants, and practice managers) working within general practices with experience of implementing and/or delivering a group consultation were eligible to participate. A list of eligible participants was sought from NHS England and their national group consultation training provider, and invited to participate via email.

With written consent, semi-structured telephone interviews were conducted, digitally recorded, and transcribed verbatim. Data were collected from October to December 2019 by two authors, neither of whom had prior clinical experience of group consultations. A purposive sampling approach was used to recruit participants from a range of professional backgrounds. Ethical approval was sought to conduct up to 20 interviews as this was considered a pragmatic sample size, likely to achieve theoretical saturation.^16^ Data regarding practice characteristics, geographical region, professional background, and experience of conducting group consultations were collected. There was no prior relationship between the research team and any of the participants.

Data analysis was based on the principles of the Framework Method,^17^ using Normalisation Process Theory (NPT) as an underpinning theoretical approach.^18^ NPT identifies, characterises, and explains key mechanisms that promote and inhibit the implementation, embedding, and integration of complex interventions,^19^ and was considered an appropriate lens through which to evaluate the implementation of group consultations. The constructs of NPT are:

- coherence, the sense making that people do to give meaning to an intervention or new practice;
- cognitive participation, the relational work that people do to develop a community of practice around that intervention;
- collective action, the operational work done to establish the intervention to be adopted; and
- reflexive monitoring, the appraisal work done to assess and understand the benefits and costs of implementation.

An inductive thematic analysis approach was taken before applying theory, which allowed themes to be developed from the data, and ensured important aspects of data were not missed.^20^ After a period of familiarisation, independent double coding of all transcripts was completed using NVivo (version 11). Coding was compared and revised. Subsequently, links with NPT were discussed. Two further iterative rounds were undertaken to refine the themes. Dedicated analysis meetings took place with the broader study team and facilitated a critical exploration of the dataset (including field notes), discussion on the application of NPT, review of deviant cases, and agreement on themes. Themes were subsequently mapped to NPT constructs. A final coding framework was discussed and refined with the broader study team.

## RESULTS

**Figure 1.**
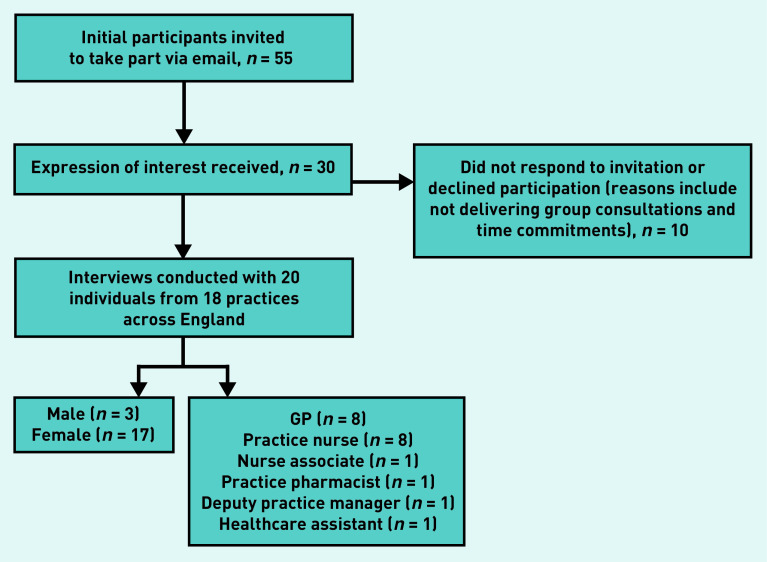
*Flowchart illustrating recruitment to study.*

Twenty multidisciplinary professionals (eight GPs, eight PNs, one nurse associate, one pharmacist, one deputy practice manager, and one healthcare assistant) participated in an interview (Figure 1). Participants (17 females, three males) were recruited from 18 practices across six regions in England. Table 1 describes practice characteristics.

**Table 1.** Practice characteristics

**Practice number^a^**	**Number of patients registered**	**Self-reported description of catchment area**	**Region**
1	65 000	Moderate deprivation, semi-rural	North West
2	18 500	Average, semi-rural	South East
3	7000	Mixed	North West
4	11 000	Average, high proportion of over-70s, lots of over-65s, lots of young families and children	South East
5	16 000	Deprived area, inner city	London
6	≥10 000	Average, mixed	Yorkshire & Humber
7	12 000	Average, suburb	London
8	15 000	Moderate deprivation	London
9	≥10 000	High levels of deprivation	West Midlands
10	16 000	Older demographic with pockets of deprivation, suburban	South East
11	10 000	High levels of deprivation	North West
12	11 000	Small town	West Midlands
13	2500	Very deprived, inner city	London
14	12 000	Suburban, middle class with patches of deprivation	South East
15	16 000	Average (no deprived wards, no affluent wards) suburb	Yorkshire & Humber
16	12 000	Both very deprived and very affluent wards, inner city	Yorkshire & Humber
17	1300	Inner city	South West
18	8000	Inner city	London

a *Practice numbers have been randomly assigned and do not relate to participant number.*

Interviews were conducted with all 20 individuals who agreed to participate. Although ethical approval permitted 20 interviews, theoretical saturation was considered to be achieved by the fifteenth interview. Interviews lasted between 23–70 minutes. Eleven participants reported having embedded group consultations in their practice, and nine reported having tried the approach and stopped delivery either temporarily or permanently. Participants reported conducting group consultations for diabetes, cancer care, chronic pain, asthma, chronic obstructive pulmonary disease, rheumatoid arthritis, depression, menopause, pre-diabetes, hypertension, and cardiovascular risk.

All inductive codes and themes could be mapped to the four constructs of NPT (Supplementary Table S1); however, different theme names were selected to best represent the findings: sense making of group consultations (coherence); the work associated with initiating group consultations (cognitive participation); the experiences of operationalising group consultations (collective action); and sustaining change (reflexive monitoring). These are presented below. Because of the risk to anonymity, the authors have not reported professional background alongside quotes.

### Sense making of group consultations

The ways in which participants made sense of the idea of group consultations related to several drivers and motivators. A common perception was that the current way of working in primary care was unsustainable due to staff ‘burn-out’ and increasing demands on services, and that group consultations had the potential to relieve strain on services by reducing workload burden and increasing capacity (freeing up appointment time). Acknowledging an initial increase in work was likely; some participants believed that this would release capacity in the long term. Other motivating factors for establishing group consultations included innate drivers, personal interest in a condition being managed by the approach, and appealing to clinicians’ sentiment of care to improve the management and outcomes of LTCs:

*‘I am absolutely convinced this is the way that it’s* [LTC management] *got to go. There’s no way we’re going to reverse this issue with heart disease, depression and all of these conditions without these sorts of tools.’*

(Participant [P]10)

Despite many participants describing positive perceptions about the approach, several examples of staff resistance, hesitancy, and, as one participant described it, *‘jitteriness’* (P17), were reported.

These were suggested to be due to: uncertainty regarding the approach itself or their role in implementing or delivering group consultations; individual beliefs about their capability or capacity to undertake group consultations and whether this was their responsibility; or the desire to adopt new roles and responsibilities:

*‘You have huge imposter syndrome, I can’t do this, I’m not trained to do this.’*

(P2)

*‘They* [staff] *don’t really fancy standing up in front of a group of people and testing themselves if you like in front of that group to the point that, am I going to be able to answer all of this without my usual computer to reference and, all my usual things that I can go to for help. So there’s that sort of nervousness.’*

(P6)

### The work associated with initiating group consultations

Participants described how the planning, initiation, and implementation of group consultations was often dependent on a group consultation ‘champion’, identified as an individual who voluntarily adopted leadership and the role of influencer to drive group consultations within their practice. Champions were typically GPs or PNs with an interest in either the approach itself or a condition to be managed by the approach. Champions were often supported by ‘professional launch pads’ (incentives from a range of funding streams and schemes such as fellowships or financial initiatives) to undertake the work associated with initiating group consultations. Champions sought to engage decision makers, patients, colleagues, and clinical commissioning groups (CCGs) (clinically led statutory NHS bodies responsible for the planning and commissioning of healthcare services for their local area), yet many spoke of the difficulties in influencing colleagues and key decision makers to ‘buy-in’ to the approach. Engagement ranged from ‘passive support’, whereby individuals did not impede the set-up of group consultations yet did not actively engage with the process (reportedly because of competing priorities or reluctance to change), and ‘blockers’, whereby the attitudes and behaviours of certain individuals could slow or halt momentum if they did not wish to engage in implementation or delivery:

*‘The pilot was very much driven by us, the GPs and management sort of allowed us to run with it … but we didn’t get masses of time to do it … And we didn’t get much admin back-up, so it was quite a struggle.’*

(P3)

*‘They weren’t against me doing it, but it was very definitely “Well you can do this because we’re not quite sure”.’*

(P8)

*‘I had a number of blockers including my own partners who just saw it as one of my schemes and let me carry on and do it … the nurse who was also involved — she didn’t believe in it … she managed to delay it and delay and delay the first and only one that we’ve had.’*

(P1)

One participant described how one of their colleagues had influence over other staff and *‘kiboshed’* the others and put a *‘big brake’* (P1) on implementation. Findings suggest that, in these instances, the champion had to dedicate more time to gaining support or engagement to implement the approach.

Whole-practice engagement was critical to implementation success to ‘sell’ the approach to staff and patients to optimise buy-in and patient attendance. The importance of *‘good working structures and relationships’* and *‘regular meetings’* (P10) for instigating change within a practice was identified:

*‘Get the decision makers involved because if they’re not involved, they will stop you, you know, passively, if not actively, because all they need to do is push it to the curb and people’s enthusiasm goes. Because particularly clinicians, you know, life’s busy and unless you’ve got some space to be able to keep pushing, keep pushing, it’s difficult to keep things going.’*

(P1)

Practice-level contextual barriers to implementation included IT systems for embedding and organising appointments, practice culture, competing interests, and access to a dedicated, trained facilitator.

Participants reported underestimating the workload required to initiate group consultations while maintaining their day job. Additional work included project management, marketing, reviewing business models, and contractual agreements:

*‘It feels to me like we’re trying to change the wheels of a Formula One car during the race and we have, we have to keep everything else going whilst trying to support this.’*

(P10)

### The experiences of operationalising group consultations

To operationalise group consultations within a practice, instances of ‘trial and error’ and learning on the job, with a lack of planning procedure or defined roles and responsibilities, were described.

The facilitator role was described as a bespoke role that was key to the success of group consultations due to the specific skill set required. Experience in behaviour change techniques or health coaching, delivering health classes, confidence, organisation, and presentation skills were identified as important characteristics of a successful facilitator. Challenges associated with the facilitator role included: defining the role; recruiting a facilitator; a lack of interest from staff in becoming a facilitator (often due to confidence); and the amount of support required by more experienced staff to ensure facilitators were confident in the role. It was also suggested that the facilitator encountered high variability of work and the largest workload associated with operationalising, organising, and managing the approach on a day-to-day basis (for example, ensuring all staff are referring patients to the group and selling the approach, calling patients, managing group set-up, completing pre-checks, and gathering questions from the group). When asked about the facilitator, one participant stated, *‘without her I couldn’t have done it’* (P12), while another described the importance of having a *‘full-time facilitator who does it really well’* (P10) to get group consultations as the main offering for an annual review:

*‘As a facilitator, you do more of the work, you’re doing the bloods, you’re doing all the base lines then you are setting it all up ready for the clinic so that in itself is a big role because when it comes to the clinic you are at the clinic, you are doing it, you are there for that hour and the nurse comes in or the ANP* [advanced nurse practitioner] *comes in and does the talk and goes over the results but really, if you think about it, you’ve done the main bulk of the role.’*

(P19)

In some instances, GPs led and facilitated group consultations for patients with diabetes, even though diabetes management was largely PN led. This was suggested as an initial solution to initiate group consultations in their practice; however, those individuals spoke of their continued involvement either because they were one of few people interested in group consultations or that had the confidence in facilitation.

Once challenges were overcome, group consultations were reported to mitigate the problems of individual consultations, because of both actual and perceived extra time, resulting in staff feeling more relaxed and to *‘bring the joy back into consultations’* (P15). In contrast, individual consultations were described as monotonous, isolating, and better suited to acute care, with pressure to get patients *‘out of the door’* (P8) due to time and workload demands:

*‘The time pressure’s off, you’re not looking at the clock and thinking, oh God I’m running late, you’re inevitably running late on one-to-ones. Whereas group consults you’re not because you’ve got your half hour or whatever it is, so it’s so relaxing and empowering … it’s just a nicer experience.’*

(P18)

Participants perceived a shift of agenda and power by suggesting that individual appointments have a clinician-led agenda and tick box mentality (driven by policy drivers and financial incentives such as the Quality and Outcomes Framework [QOF]), whereas group consultations were perceived as being driven and led by patients. The group dynamic was suggested as the most powerful benefit of group consultations. For example, by peer support from patients *‘challenging each other in a way that staff can’t’* (P15) regarding sensitive issues such as diet and exercise:

*‘For example, “Why on earth are you eating that, you know it’s gonna put your sugar up, why are you doing that?”’*

(P15)

Group consultations were described to improve team cohesiveness, enhance knowledge and capability of junior clinicians, and create an opportunity for staff to learn clinical knowledge and skills, facilitation, and presentation skills:

*‘Our in-house pharmacist learnt a lot from listening to the asthma specialist. And then used that information when she does one-to-one consultations and also in the reviews from patients who have been in the group consultations and I think in a way we’ve all learnt together. It’s been an educational activity for the staff, the professional staff as well.’*

(P16)

### Sustaining change

Participants described the ongoing work involved in finding the right path for the routinisation of group consultations, and the need for support with the significant administrative and organisational burden. Difficulties in making group consultations *‘business as usual’* (P14) included a lack of implementation support and funding, the wrong time to be taking on a new project, vacancies in the team, practice pressures, and insufficient resources for ongoing clinical and professional support (such as training, mentoring, peer debriefing, and planning meetings). Competing priorities and drivers, along with an inability to convince decision makers, were reported reasons for unsuccessful routinisation of the approach:

*‘We are not getting them embedded to the extent that I would like. It’s a huge frustration for me and that’s happening because we’re struggling to get the senior buy-in. Not because they’re not — I mean they’re letting us do it. They’re not against it, but there are a lot of competing priorities and it’s a struggle to get this to be the priority that people then put the effort into getting the embedding happening.’*

(P8)

Participants who worked in a practice that had embedded group consultations spoke of how they were able to, or were planning to, scale out the model to other conditions or settings (for example, schools for paediatric asthma) and how this may improve multidisciplinary integrated care working.

Despite several practices delivering group consultations for ≥18 months, many reported it to be too soon to measure the impact on practice capacity because of a lack of evaluation data. Factors affecting evaluation included the ability to select and collect process outcomes, and clarity as to whose responsibility it was to evaluate. In contrast, most participants spoke of the collection and often improvement of clinical outcome measures such as weight loss, biomarkers for patients with diabetes, and reduced prescribed medication.

At a systems level, participants highlighted a lack of flexibility in terms of CCG support and how tariff and payment systems were not structured to recognise group consultation activity for payment. As well as resulting in reduced resource, this was demoralising:

*‘It became people doing things in their own time for free because the CCG didn’t count for it at the initial stages. Why wait until you’ve proven a concept and got it just right before you pay staff to do it, why don’t you count it for activity, because then it might actually happen. So I think if the CCG were more supportive, and for me, I felt a bit upset by that, I thought well actually, we’re doing all this hard work to develop and grow primary care, and to make a real difference and to have impact and to make this change happen, the CCG should be celebrating, they should be joining in and they should be behind it.’*

(P14)

## DISCUSSION

### Summary

This novel qualitative interview study has utilised an implementation theory (NPT) to understand the dynamics of implementing and embedding group consultations (a complex intervention) in UK primary care. This study is the first, to the authors’ knowledge, to explore the experiences of staff from general practices in England in implementing and delivering group consultations. Findings illustrate that clinicians enjoyed group consultations because of enhanced multidisciplinary working and the ability to provide more patient-centred care. However, a range of motivators influence the engagement, or coherence, of the approach by practice staff. Factors required to initiate (cognitive participation) and sustain group consultations include clear leadership (from a group consultation champion), an implementation plan at the outset, clarity regarding roles and responsibilities, and wider support from policy mechanisms (tariffs, for example) and CCGs. Findings highlight the significant workload associated with initiating and embedding group consultations, and the need to measure this locally and nationally to inform future implementation and delivery.

### Strengths and limitations

Strengths of this study include the multidisciplinary mix of general practice staff from a wide geographical spread and a robust approach to data analysis, including double coding, which enhances the trustworthiness of the findings. Furthermore, the involvement of a multidisciplinary stakeholder group, including patient representatives, informed topic guide development and ensured the research addressed questions pertinent to stakeholders. A further strength to this work is the use of NPT to capture the complexities involved in implementation, and help to explain how participants understand, engage with, reflect on, and evaluate the implementation of a new practice.^21^

A potential limitation to the transferability of the study is the recruitment of participants via one group consultation training provider; clinicians trained by an alternative provider may have different experiences. However, the authors believe that the inclusion of practices from six regions of England, with a broad range of practice characteristics, aids transferability to UK general practice. The study sample, predominantly females, all having received training from the same provider, may risk bias or have encouraged participation from those with more positive views towards group consultations.^6^ However, the findings did represent a breadth of views, including the identification of many challenges and reasons why delivery of group consultations had ceased. The range of interview duration has the potential to affect the data, yet this reflects the challenges of interviewing primary care staff, and, despite this, theoretical saturation was achieved. Because of confidentiality and the small numbers of certain professional groups (one pharmacist, healthcare assistant, and practice manager), the authors were unable to report the link between participant quotes and professional background. To better understand the potentially differing views of staff from different professional backgrounds, further studies including more interviews with these professional groups may be needed.

### Comparison with existing literature

Several motivators for engaging in the implementation and delivery of group consultations were identified. Using the lens of NPT, this relates to coherence (sense making) and how individuals and practices understood the components of group consultations and how they differ from current practice; developed a shared understanding of the aims and benefits of group consultations; and recognised individual responsibilities concerning group consultations and their potential value. For example, those who held the perception of ‘invest to save’, whereby a greater time commitment was required in the early stages of implementation but would reduce as the model became embedded, were typically bought in to the beneficial ways that group consultations could influence practice. On the other hand, hesitancy or reluctance to implement group consultations made engagement work (or cognitive participation) challenging, meaning that group consultation champions had to dedicate significant time to the relational work needed to increase the likelihood of achieving and sustaining implementation.

This study illustrates the important role of champions in encouraging engagement of the whole practice team. This aligns with the cognitive participation construct of NPT, which describes the work needed to initiate a new set of practices, and how key individuals are driving them forward while ensuring the involvement of others. A systematic review of reviews exploring the causes of the implementation gap identified how influential champions who were respected and trusted by staff to drive change had a positive impact on implementation.^22^ A recent qualitative study by the authors,^23^ exploring the uptake of a model of care for osteoarthritis in UK primary care, found that profession, project, and patient-specific champions were instrumental in implementation and enabling change, although a clearer definition of the role is needed to promote consistency and increase visibility.

Participants enjoyed delivering group consultations for reasons consistent with other studies, for example: multidisciplinary learning (including staff learning from patients);^6^^,^^24^ the opportunity to up-skill; improved team cohesiveness;^10^ and, a less time-pressured consultation that supports LTC management.^7^ For several participants, these factors built and maintained confidence in the approach, which is consistent with the collective action construct of NPT. Group consultations, therefore, may offer a potential solution for addressing issues with staff morale, burnout, and job satisfaction, while supporting patient-centred care. This supports findings from Kirsh and colleagues,^6^ who undertook a realist review of 71 high-quality primary research studies to build a conceptual model of the group consultation approach, and identify nine overarching mechanisms to explain how group consultations work. The themes identified from professionals are consistent with the literature regarding patients who have participated in group consultations, which has shown positive data relating to the patient–clinician dynamic, improved satisfaction, and a perception that staff appear less hurried.^12^

Facilitators to initiating and embedding group consultations include clear leadership (by a champion), clarity regarding facilitator roles and responsibilities, wider organisational buy-in (CCG, for example), and a continual investment of staff working collectively to implement the approach. Externally sourced funding or protected time was often required to support the workload associated with the relational and operational work required (cognitive participation and collective action) within a practice. The facilitator role is multifaceted and was shown to enhance collective action and the ‘doing’ phase of implementation (including communicating with practice staff to maintain confidence in the approach, and organising processes and systems within a practice). Findings illustrated that the often-underestimated workload, including planning and preparatory work, often fell to one or two individuals who assumed responsibility for driving forward implementation within a practice. In some instances, the reliance on these individuals and lack of whole-practice engagement meant that the approach failed to become embedded.

Many of the present study findings relate to the challenges of implementation and sustaining change. Participants identified that specialist implementation expertise, training, and, in some cases, funding were required to support the process and facilitate routine embedding. Difficulties obtaining buy-in from senior decision makers were described, as also demonstrated elsewhere by the present authors,^23^ whereby PNs often lacked the capacity or autonomy to implement change, despite having the ambition to do so as discretion over decision making fell to the practice manager or GP partners. Further challenges related to the difficulties in recruiting, selecting, and training a facilitator due to poor definition and understanding of the role. In some cases, GPs themselves facilitated the groups (because of lack of engagement or confidence of other staff), which subsequently increased their responsibilities.

Issues relating to the evaluation and appraisal of group consultations influenced the sustainability (reflexive monitoring) of the approach. There were few instances where general practice staff collectively agreed on the effectiveness and usefulness of the approach. Some participants did not consider group consultations to have been ‘successfully’ embedded within their practice or sustained long enough to consider a robust evaluation of time or cost savings. Several studies have explored health system costs, yet cost–benefit concerns remain, and evidence to date is equivocal.^10^^–^^12^ There appeared to be a greater focus on collecting clinical outcomes rather than process measures, and participants described a lack of capacity or resource to analyse and share the findings more broadly. When asked, participants used hopeful language to report the ‘anticipated’ or ‘predicted’ benefits of the approach (cost or time saving), even though that had not been demonstrated.

### Implications for practice and research

Several implications for practice and research have been identified. First, the authors suggest close attention is paid to implementation planning with an implementation strategy that considers contextual circumstances (including identifying leadership, a champion, and clarifying roles and responsibilities), and local evaluation data to support the set-up and sustainability of this model of care. Second, to provide an additional lever for adoption, innovations need to be recognised through payment mechanisms and relevant data collected to support national policy on reducing inequalities in access to general practice services.^25^^,^^26^ Given the workload associated with implementation, evaluation of process outcomes is required locally and nationally. Data regarding the numbers of patients offered a group consultation, uptake, demographics, cost, resource use (such as time taken to prepare for a group consultation and complete notes compared with one-to-one appointments), and broader healthcare utilisation (for example, reduced consultations or hospital admissions) would generate practice-based learning to inform the implementation, operationalisation, and sustainability of the approach, relevant to all key stakeholders (including policymakers and commissioners). This may inform the need for and magnitude of initial investment needed to launch group consultations. Finally, primary care networks may wish to consider the ‘specialist’ facilitation of groups (across their footprint) to support services evolving from secondary care into the community.

In view of the changes in healthcare delivery that have ensued during the COVID-19 pandemic, further research is now needed to explore the acceptability, feasibility, and effectiveness of online group consultations. Despite a plethora of recent studies exploring virtual consultations, most research to date has focused on individual consultations rather than virtual group consultations (VGCs).^27^^–^^30^ However, a recent systematic review to determine the feasibility, acceptability, effectiveness, and implementation of VGCs reported that the use of video was broadly acceptable, had significant IT challenges — albeit that could be overcome — and that the visualisation of the patient home could facilitate more context-specific support.^31^ However, in common with studies of face-to-face consultations, studies of acceptability of VGCs do not illuminate barriers to patient perception, and do not help to understand if this approach to health care widens access, or, instead, promotes health inequality by excluding those without skills or confidence to participate in online or physical groups. Therefore, the extent to which the characteristics of the patient population (for example, health and digital literacy, and sociodemographic status) affects the uptake and effectiveness of both face-to-face and VGC delivery remains a further important unanswered question.

This qualitative study has explored the experience of implementing and delivering group consultations in general practice in England from the perspective of the workforce. Using the lens of NPT, this study has identified a range of individual motivators that influence staff engagement with implementation and delivery of the approach. Clinicians enjoyed group consultations; however, a significant amount of work is required to initiate and sustain the approach. To facilitate this, an implementation plan is recommended at the outset along with strong leadership, clear roles and responsibilities, and wider organisational support. Further research and practice-based evaluation is needed to capture and better understand process outcomes.
